# Old age is associated with worse treatment outcome and frequent adverse drug reaction in *Mycobacterium avium* complex pulmonary disease

**DOI:** 10.1186/s12890-022-02063-2

**Published:** 2022-07-14

**Authors:** Joong-Yub Kim, Na Young Kim, Hee-Won Jung, Jae-Joon Yim, Nakwon Kwak

**Affiliations:** 1 Division of Pulmonary and Critical Care Medicine, Department of Internal Medicine, Seoul National University Hospital, Seoul, Republic of Korea; 2 Present Address: Division of Pulmonary, Allergy, and Critical Care Medicine, Department of Internal Medicine, Hallym University Dongtan Sacred Heart Hospital, Hwaseong, Gyeonggi-do Republic of Korea; 3 Division of Geriatrics, Department of Internal Medicine, Asan Medical Center, University of Ulsan College of Medicine, Seoul, Republic of Korea; 4 Department of Internal Medicine, Seoul National University College of Medicine, Seoul, Republic of Korea

**Keywords:** Aging, Antibiotic therapy, Frailty, Geriatric, Nontuberculous mycobacteria

## Abstract

**Background:**

The number of patients with nontuberculous mycobacterial pulmonary disease (NTM-PD) is rapidly increasing globally, especially in the older population. However, there is a dearth of evidence regarding the impact of aging on the treatment outcomes of NTM-PD.

**Methods:**

We analyzed consecutive patients who satisfied the diagnostic criteria for *Mycobacterium avium* complex (MAC)-PD and received antibiotic treatment between January 2009 and December 2020 at a tertiary referral hospital in Korea. The main outcomes were (1) long-term treatment success, defined by negative culture conversion for more than 12 months; and (2) adverse drug reactions (ADRs). Multivariable logistic regression model was used to evaluate the association between age and main outcomes.

**Results:**

A total of 614 patients (median age, 65 years, interquartile range [IQR] 57–73 years; men, 35.3%) were included. Median treatment duration (530 days, IQR 290–678 days; *P* for trend < 0.001) and long-term treatment success (*P* for trend = 0.026) decreased, whereas ADRs (*P* for trend < 0.001) increased significantly with age. Multivariable analyses demonstrated that age ≥ 80 years was an independent factor associated with ADRs (adjusted odds ratio [aOR] 3.29; 95% confidence interval [CI] 1.05–10.28) and worse treatment outcome (aOR 0.42; 95% CI 0.19–0.91).

**Conclusions:**

Aging is associated with worse treatment outcome and frequent ADRs of patients with MAC-PD. Individualized treatment with reduced-intensity may be a reasonable alternative for older adults.

**Supplementary Information:**

The online version contains supplementary material available at 10.1186/s12890-022-02063-2.

## Background

Nontuberculous mycobacteria (NTM), comprising more than 200 mycobacteria other than *Mycobacterium leprae* and *M. tuberculosis* complex, are ubiquitous organisms that can be isolated from soil, dust, and municipal water. The most common manifestation of NTM infection is pulmonary disease (PD), and the burden of NTM-PD has increased globally [1]. Population-based studies have reported rising incidence and prevalence of NTM-PD in the United States and other developed countries, in those with underlying chronic lung disease and older populations. In South Korea, during the last decade, the prevalence of NTM-PD has increased more than four times, and the overall prevalence has increased with age [2]. With its substantial morbidity and mortality, NTM-PD is becoming a significant healthcare burden in older adults [3, 4].

The mainstay of treatment for NTM-PD is long-term antibiotic therapy, which generally requires the combination of at least three antibiotics for more than 12 months after culture conversion [1, 5]. This multidrug treatment results in adverse drug reactions (ADRs) in most patients [6], which interrupts the treatment in 17–75% of patients [7]. According to a retrospective study in South Korea, 112 of 295 (38.0%) patients had discontinued treatment for *M. avium* complex (MAC)-PD owing to ADRs within a year [8].

ADRs associated with NTM-PD treatment can be more problematic among older patients. Adding long-term treatment for NTM-PD in these patients may exacerbate the adverse health effects of polypharmacy and drug–drug interactions since they are already on multiple medications for underlying comorbidities [9, 10]. As NTM-PD is considered a condition associated with immunosenescence [11], decreased physiological reserve and increased vulnerability to potential stressors with frailty in these populations could be especially problematic [12–15]. Frequent ADRs tied to treatment for NTM-PD lead to early discontinuation of treatment, dosage modification, increased hospitalization, lasting morbidity, and ultimately, mortality [1, 16, 17]. Because the reported overall treatment success rates for NTM-PD are as low as 33.0–60.0% depending on the causative species [18, 19], clinical decision between the potential benefit of antibiotic treatment and harm from ADRs could be tailored in older patients. Unfortunately, very little research has been done on the clinical relevance of aging and frailty in the treatment of NTM-PD, and current guidelines do not provide specific recommendations for different age groups [5].

The purpose of this study was to examine age-based differences in treatment patterns, outcomes, and ADRs in patients with MAC-PD, the most common species of NTM worldwide [20]. We aimed to determine whether age increases the risk of treatment failure and ADRs.

## Methods

### Study design and patient selection

We retrospectively analyzed patients (1) aged 18 years or older, (2) who met the diagnostic criteria of the American Thoracic Society, the European Respiratory Society, the European Society of Clinical Microbiology and Infectious Diseases, and the Infectious Diseases Society of America for NTM-PD [5], (3) had MAC species identified from respiratory samples to be the causative strain, and (4) had initiated antibiotic treatment targeting for MAC-PD between January 1, 2009, and December 31, 2020, at Seoul National University Hospital, a tertiary referral, academic center in South Korea. When patients underwent multiple treatment trials, only the first trial was included in the analysis. Patients were excluded if treatment was initiated at another hospital or treatment was not initiated during the study period. Some patients in this study were included in our previous reports [21, 22]. The study protocol was approved and the need to obtain informed consent was waived by the institutional review board at Seoul National University Hospital (Approval No. H-2108-084-1245). The study was performed in accordance with the principles of the Declaration of Helsinki.

### Data collection

The demographic and clinical characteristics of the patients at the start of antibiotic treatment were collected. The variables included age, sex, body mass index (BMI), presence of cavitary lesions on thoracic computed tomography, erythrocyte sedimentation rate (ESR), acid-fast bacilli smear results, mycobacterial culture findings, drug susceptibility test results, history of pulmonary tuberculosis, and the presence of other comorbidities determined by physicians’ diagnosis (bronchiectasis, lung cancer, other malignancies, chronic liver diseases, chronic kidney diseases, cerebrovascular diseases, dementia, chronic heart failure, and myocardial infarction). The BACES score [22] and Charlson comorbidity index [23] were calculated for each patient. Details on the treatment regimen, treatment duration, and subsequent mycobacterial culture results were collected.

### Outcomes definition

The primary outcome was long-term treatment success, defined as satisfying the negative culture conversion criterion without disruption by positive cultures for more than 12 months [18]. Negative culture conversion was defined as three or more consecutive negative mycobacterial cultures collected at least 4 weeks apart [24]. Another main outcome was ADRs, defined as medically significant or life-threatening events [25] that require discontinuation of treatment, typically grade 3 or more according to the Common Terminology Criteria for Adverse Events, version 5.0 [26]. If a drug was suspected to be the culprit, only the drug was halted; otherwise, the entire treatment was stopped. ADRs were organized according to the system organ classes of the Medical Dictionary for Regulatory Activities [27], and culprit drugs, which were determined by physicians on duty, were investigated. When deaths occurred during treatment, electronic medical records, if available, were examined to evaluate possible association with ADRs and treatment outcomes were assessed based on the most recent mycobacterial culture results.

### Statistical analysis

For the analysis, patients were divided into five age groups according to their age at treatment initiation: < 50 years, ≥ 50 to < 60 years, ≥ 60 to < 70 years, ≥ 70 to < 80 years, and ≥ 80 years [28]. Demographic and clinical characteristics, treatment regimens, outcomes, and ADRs were summarized as counts and proportions for categorical variables and medians with interquartile ranges for continuous variables. To assess the difference in the proportion and distribution of variables by age group, *P* for trend was calculated using generalized linear regression. Multivariable logistic regression analysis was conducted to evaluate whether old age was independently associated with ADRs and long-term treatment success. Clinically meaningful variables were selected a priori for multivariable analysis based on a literature review [29, 30]. Two-sided *P* values < 0.05 were considered statistically significant. All analyses were conducted using R software (version 4.1.1; R Foundation for Statistical Computing, Vienna, Austria).

## Results

### Patient characteristics

A total of 614 patients who received treatment for MAC-PD during the study period were identified. The median age was 65 years (interquartile range [IQR], 57–73 years), and 217 (35.3%) patients were men. Among them, 54 patients (8.8%) aged < 50 years, 138 patients (22.5%) aged ≥ 50 to < 60 years, 184 patients (30.0%) aged ≥ 60 to < 70 years, 185 patients (30.1%) aged ≥ 70 to < 80 years, and 53 patients (8.6%) aged ≥ 80 years. The proportion of men (*P* for trend < 0.001) and patients with BMI < 18.5 kg/m^2^ (*P* for trend = 0.028) increased with age. As expected, ESR also increased with age (*P* for trend < 0.001) [31]. The presence of cavitary lesions and mycobacterial culture positivity remained similar across age groups, whereas clarithromycin resistance decreased with age (*P* for trend = 0.044). With regard to comorbidities, the proportion of patients with interstitial lung disease (*P* for trend = 0.010), malignancy other than lung cancer (*P* for trend = 0.010), cerebrovascular disease (*P* for trend = 0.003), and dementia (*P* for trend = 0.020) increased in the older age groups. Detailed demographic and clinical characteristics of the age groups are summarized in Table 1.

**Table 1:** Baseline demographic and clinical characteristics by age groups

Characteristics	Total	age < 50	≥ 50 age < 60	≥ 60 age < 70	≥ 70 age < 80	age ≥ 80	*P* for trend
	(N = 614)	(n = 54)	(n = 138)	(n = 184)	(n = 185)	(n = 53)	
Age, years (IQR)	65.0 (57.3–73.2)	44.9 (41.2–47.7)	54.7 (52.4–57.4)	63.8 (61.4–66.4)	73.6 (71.5–75.7)	81.6 (80.4–83.6)	< 0.001
Men, n (%)	217 (35.3)	13 (24.1)	24 (17.4)	63 (34.2)	90 (48.6)	27 (50.9)	< 0.001
BMI, kg/m^2^ (IQR)	20.3 (18.3–21.9)	19.9 (18.3–21.0)	20.6 (18.8–21.7)	20.5 (18.8–22.0)	20.1 (17.6–22.2)	19.6 (17.4–22.1)	0.41
< 18.5 kg/m^2^, n (%)	154 (26.8)	13 (27.1)	28 (21.9)	36 (20.9)	61 (34.9)	16 (30.8)	0.028
Cavitary lesion, n (%)	283 (46.1)	21 (38.9)	65 (47.1)	81 (44.0)	84 (45.4)	32 (60.4)	0.21
ESR, mm/hr (IQR)	25.0 (13.0–47.0)	21.0 (10.5–35.5)	20.0 (11.0–37.0)	23.0 (11.0–45.5)	30.0 (18.0–54.0)	51.5 (27.0–75.0)	< 0.001
Elevated ESR, n (%)	291 (59.3)	25 (53.2)	50 (47.2)	82 (54.3)	101 (67.8)	33 (86.8)	< 0.001
Positive mycobacterial culture at treatment initiation, n (%)	497 (80.9)	44 (81.5)	107 (77.5)	148 (80.4)	152 (82.2)	46 (86.8)	0.66
Clarithromycin resistance, n (%)	11 (2.7)	2 (5.9)	6 (6.6)	1 (0.8)	2 (1.7)	0 (0.0)	0.044
BACES score (IQR)	2 (1–3)	1.0 (1.0–2.0)	1.0 (1.0–2.0)	2.0 (1.0–3.0)	3.0 (2.0–4.0)	3.0 (3.0–4.0)	< 0.001
*Comorbidities*							
History of tuberculosis, n (%)	78 (12.7)	8 (14.8)	16 (11.6)	21 (11.4)	23 (12.4)	10 (18.9)	0.65
Chronic obstructive pulmonary disease, n (%)	16 (2.6)	0 (0.0)	0 (0.0)	6 (3.3)	8 (4.3)	2 (3.8)	0.096
Bronchiectasis, n (%)	203 (33.1)	22 (40.7)	43 (31.2)	64 (34.8)	64 (34.6)	10 (18.9)	0.14
Interstitial lung disease, n (%)	17 92.8)	1 (1.9)	3 (2.2)	1 (0.5)	7 (3.8)	5 (9.4)	0.010
Lung cancer, n (%)	11 (1.8)	0 (0.0)	1 (0.7)	3 (1.6)	5 (2.7)	2 (3.8)	0.41
Other malignancy, n (%)	81 (13.2)	5 (9.3)	16 (11.6)	26 (14.1)	19 (10.3)	15 (28.3)	0.010
Diabetes, n (%)	38 (6.2)	1 (1.9)	6 (4.3)	8 (4.3)	18 (9.7)	5 (9.4)	0.072
Chronic liver disease, n (%)	36 (5.9)	1 (1.9)	7 (5.1)	9 (4.9)	12 (6.5)	7 (13.2)	0.12
Chronic renal disease, n (%)	13 (2.1)	0 (0.0)	0 (0.0)	5 (2.7)	5 (2.7)	3 (5.7)	0.092
Cerebrovascular disease, n (%)	33 (5.4)	0 (0.0)	1 (0.7)	10 (5.4)	17 (9.2)	5 (9.4)	0.0030
Dementia, n (%)	12 (2.0)	0 (0.0)	0 (0.0)	2 (1.1)	7 (3.8)	3 (5.7)	0.020
Charlson comorbidity index, median (IQR)	3.0 (2.0–4.0)	1.0 (0.0–1.0)	2.0 (1.0–2.0)	3.0 (2.0–3.0)	4.0 (3.0–5.0)	5.0 (4.0–7.0)	< 0.001

Lung cancer and other malignancy refer to pathologically proven cancer of any stage diagnosed before the treatment of MAC-PD, regardless of current activity

*IQR* interquartile range, *BMI* body mass index, *ESR* erythrocyte sedimentation rate, *BACES* body mass index, age, cavity, erythrocyte sedimentation rate, sex

### Treatment regimen and outcome

Treatment regimens were administered in accordance with international treatment guidelines [1, 5]: a macrolide was administered in combination with one or more of ethambutol, rifampicin, clofazimine, amikacin, or streptomycin. Along with the total median number of drugs, which was three, the proportion of patients who received parenteral drugs remained similar across age groups (Table 2). The median duration of treatment, which was 530 days in patients aged < 50 years, decreased gradually to 348 days in patients aged ≥ 80 years (*P* for trend < 0.001) (Additional file 1: Fig. S1). The median duration of parenteral drug use also decreased in the older groups (*P* for trend < 0.001). The overall long-term treatment success rate of the study cohort was 39.9% (95% confidence interval [CI], 36.0%–43.8%), which decreased significantly with age in a linear relationship as follows: 42.6% in patients aged < 50 years, 46.4% in patients aged ≥ 50 to < 60 years, 41.3% in patients aged ≥ 60 to < 70 years, 38.4% in patients aged ≥ 70 to < 80 years, and 20.8% in patients aged ≥ 80 years (*P* for trend = 0.026) (Table 3; Additional file 1: Fig. S1).

**Table 2:** Treatment regimen by age groups

Antibiotic drugs	Total	age < 50	≥ 50 age < 60	≥ 60 age < 70	≥ 70 age < 80	age ≥ 80	*P* for trend
	(N = 614)	(n = 54)	(n = 138)	(n = 184)	(n = 185)	(n = 53)	
Azithromycin, n (%)	407 (66.3)	29 (53.7)	79 (57.2)	127 (69.0)	132 (71.4)	40 (75.5)	0.008
Clarithromycin, n (%)	311 (50.7)	38 (70.4)	73 (52.9)	86 (46.7)	93 (50.3)	21 (39.6)	0.014
Ethambutol, n (%)	599 (97.6)	53 (98.1)	137 (99.3)	181 (98.4)	177 (95.7)	51 (96.2)	0.246
Rifampicin, n (%)	505 (82.2)	48 (88.9)	115 (83.3)	157 (85.3)	146 (78.9)	39 (73.6)	0.133
Clofazimine, n (%)	93 (15.1)	5 (9.3)	21 (15.2)	26 (14.1)	32 (17.3)	9 (17.0)	0.663
Parenteral drugs, n (%)	68 (11.1)	9 (16.7)	12 (8.7)	22 (12.0)	16 (8.6)	9 (17.0)	0.229
Amikacin, n (%)	43 (7.0)	3 (5.6)	9 (6.5)	13 (7.1)	11 (5.9)	7 (13.2)	0.454
Streptomycin, n (%)	31 (5.0)	8 (14.8)	3 (2.2)	12 (6.5)	6 (3.2)	2 (3.8)	0.004
Number of drugs, median (IQR)	3.0 (3.0–4.0)	3.0 (3.0–4.0)	3.0 (3.0–4.0)	3.0 (3.0–4.0)	3.0 (3.0–4.0)	3.0 (3.0–4.0)	0.426

*IQR* interquartile range

**Table 3:** Treatment duration, outcome, and adverse drug reactions (ADRs) by age groups

Outcome	Total	age < 50	≥ 50 age < 60	≥ 60 age < 70	≥ 70 age < 80	age ≥ 80	*P* for trend
	(N = 614)	(n = 54)	(n = 138)	(n = 184)	(n = 185)	(n = 53)	
Treatment duration, median (IQR)	530 (290–678)	530 (329–658)	574 (392–721)	546 (349–686.5)	457 (196–630)	348 (98–551)	0.0068
Duration of parenteral drugs, median (IQR)	59.0 (16.5–236.5)	315.0 (206.0–524.0)	109.0 (15.0–260.0)	84.0 (28.0–168.0)	28.5 (13.5–74.5)	51.0 (8.0–66.0)	0.0015
Long–term treatment success, n (%)	245 (39.9)	23 (42.6)	64 (46.4)	76 (41.3)	71 (38.4)	11 (20.8)	0.026
Total ADRs, n (%)	121 (19.7)	10 (18.5)	19 (13.8)	22 (12.0)	49 (26.5)	21 (39.6)	< 0.001
Gastrointestinal	51 (8.3)	3 (5.6)	5 (3.6)	5 (2.7)	28 (15.1)	10 (18.9)	< 0.001
Eye	31 (5.0)	1 (1.9)	8 (5.8)	8 (4.3)	10 (5.4)	4 (7.5)	0.69
Hepatobiliary	20 (3.3)	4 (7.4)	5 (3.6)	2 (1.1)	7 (3.8)	2 (3.8)	0.20
Neurological	16 (2.6)	0 (0.0)	3 (2.2)	4 (2.2)	8 (4.3)	1 (1.9)	0.42
Skin and subcutaneous	16 (2.6)	1 (1.9)	3 (2.2)	4 (2.2)	8 (4.3)	0 (0.0)	0.42
Ear and labyrinth	13 (2.1)	1 (1.9)	1 (0.7)	5 (2.7)	5 (2.7)	1 (1.9)	0.75
General	8 (1.3)	0 (0.0)	1 (0.7)	2 (1.1)	2 (1.1)	3 (5.7)	0.060
Musculoskeletal	7 (1.1)	1 (1.9)	1 (0.7)	1 (0.5)	3 (1.6)	1 (1.9)	0.80
Blood and lymphatic	6 (1.0)	1 (1.9)	3 (2.2)	2 (1.1)	0 (0.0)	0 (0.0)	0.31
Renal	4 (0.7)	0 (0.0)	1 (0.7)	1 (0.5)	1 (0.5)	1 (1.9)	0.80
Cardiac	3 (0.5)	1 (1.9)	0 (0.0)	0 (0.0)	2 (1.1)	0 (0.0)	0.26
Hypersensitive reaction	2 (0.3)	0 (0.0)	1 (0.7)	0 (0.0)	1 (0.5)	0 (0.0)	0.76
Others	16 (2.6)	0 (0.0)	1 (0.7)	3 (1.6)	7 (3.8)	5 (9.4)	0.0049
Discontinuation of all treatment due to ADRs, n (%)	56 (9.1)	7 (13.0)	7 (5.1)	13 (7.1)	17 (9.2)	12 (22.6)	0.0025

*ADR* adverse drug reactions, *IQR* interquartile range

### ADRs

**Fig. 1:**
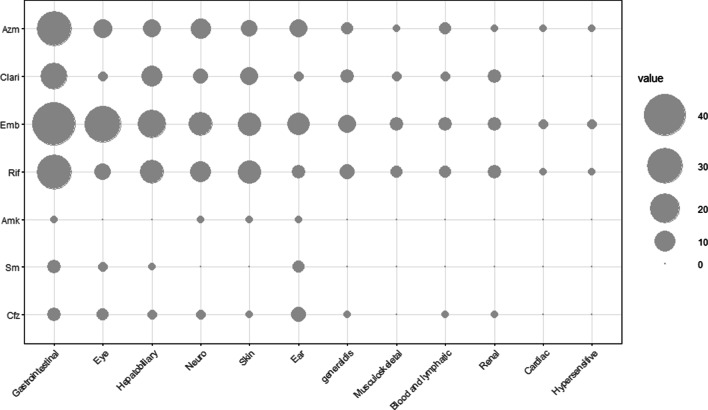
Frequency of adverse drug reactions (ADRs) by drugs. Each horizontal line refers to a drug. Each vertical line refers to a manifestation site affected by an adverse drug reaction (ADR), named according to the Medical Dictionary for Regulatory Activities system organ class. The frequency of ADRs is represented via the size of the circles. *Azm* azithromycin, *Clari* clarithromycin, *Emb* ethambutol, *Rif* rifampicin, *Amk* amikacin, *Sm* streptomycin, *Cfz* clofazimine, *Neuro* neurological, *generaldis* general disorder

At each visit, physicians performed patient interviews, physical examination, and tests for complete blood count, liver and renal function to monitor the occurrence of ADRs. The overall rate of ADRs, which necessitated the discontinuation of culprit drugs or the entire treatment, in the study cohort was 19.7% (121 patients; 95% CI 16.6–22.9%) (Table 3; Additional file 1: Fig. S1). This rate increased significantly with age from 18.5% in patients aged < 50 years to 39.6% in patients aged ≥ 80 years (*P* for trend < 0.001). The most common ADRs were gastrointestinal disorders (8.3%), followed by eye disorders (5.0%) and hepatobiliary disorders (3.3%). Gastrointestinal disorders (*P* for trend < 0.001) occurred more frequently in older age groups than that in younger age groups. There was no difference in eye, hepatobiliary, or ear disorders across age groups. Ethambutol was the most common drug associated with ADRs (82.6%), followed by rifampicin (55.4%) and azithromycin (43.8%). Parenteral drugs accounted for a relatively small number of ADRs (6.9%). The frequency of ADRs for each drug is presented in Fig. 1.

### Factors associated with ADRs

In univariable logistic regression analysis, age ≥ 80 years (odds ratio [OR] 3.04; 95% CI 1.25–7.40; *P* = 0.01), chronic renal disease (OR 4.03; 95% CI 1.27–12.72; *P* = 0.018), and chronic liver disease (OR 2.45; 95% CI 1.19–5.02; *P* = 0.015) were associated with ADRs. Multivariable analysis adjusted for sex and BMI revealed that age ≥ 80 years (adjusted OR [aOR], 3.29; 95% CI 1.05–10.28; *P* = 0.040), chronic renal disease (aOR 5.64; 95% CI 1.57–20.31, *P* = 0.0082), and chronic liver disease (aOR 3.10; 95% CI 1.24–7.79; *P* = 0.016) were independently associated with ADRs (Table 4).

**Table 4:** Multivariable logistic regression analysis of factors associated with ADRs

Characteristics	Univariable analysis OR (95% CI)	*P*	Multivariable analysis aOR (95% CI)	*P*
*Age, years*				
50	Reference		Reference	
50–59	0.71 (0.3–1.67)	0.437	0.69 (0.23–2.04)	0.501
60–69	0.59 (0.26–1.35)	0.212	0.72 (0.26–1.99)	0.524
70–79	1.58 (0.73–3.39)	0.245	2.11 (0.81–5.54)	0.128
≥ 80	3.04 (1.25–7.40)	0.014	3.29 (1.05–10.28)	0.04
Men	1 (0.65–1.52)	0.985	0.68 (0.39–1.18)	0.17
BMI	1.02 (0.95–1.09)	0.647	1.03 (0.95–1.12)	0.484
< 18.5 kg/m^2^	1.22 (0.77–1.92)	0.397		
ESR	1.01 (1–1.01)	0.099		
Elevated ESR	1.25 (0.78–2.01)	0.354		
Cavitary lesion	1.09 (0.73–1.63)	0.674		
Positive mycobacterial culture	0.97 (0.58–1.62)	0.91		
Chronic renal disease	4.03 (1.27–12.72)	0.018	5.64 (1.57–20.31)	0.0082
Chronic liver disease	2.45 (1.19–5.02)	0.015	3.1 (1.24–7.79)	0.016
Malignancy	1.49 (0.86–2.57)	0.153		
Dementia	0.35 (0.04–2.71)	0.313		
Cerebrovascular disease	0.97 (0.39–2.42)	0.943		

*OR* odds ratio, *CI* confidence interval, *BMI* body mass index, *ESR* erythrocyte sedimentation rate

### *Age* ≥ *80 years is independently associated with long-term treatment outcome*

Because ADRs increased with age, multivariable logistic regression analysis was performed to determine whether old age was independently associated with long-term treatment outcome. Even after adjusting for ADRs, age ≥ 80 years had a significant negative impact on long-term treatment success (aOR 0.42; 95% CI 0.20–0.91; *P* = 0.027), whereas ADRs did not (aOR 0.79; 95% CI 0.46–1.34; *P* = 0.377) (Table 5).

**Table 5:** Multivariable logistic regression analysis of factors associated with long-term treatment success

Characteristics	Univariable analysis OR (95% CI)	*P*	Multivariable analysis adjusted OR (95% CI)	*P*
Age ≥ 80 years	0.37 (0.17–0.80)	0.011	0.42 (0.19–0.91)	0.027
Men	0.70 (0.47–1.06)	0.091	0.72 (0.48–1.10)	0.127
BMI < 18.5 kg/m^2^	0.85 (0.54–1.32)	0.460	0.97 (0.61–1.56)	0.911
Elevated ESR	0.80 (0.54–1.18)	0.253	0.88 (0.58–1.33)	0.545
Cavitary lesion	0.79 (0.54–1.16)	0.232	0.83 (0.55–1.24)	0.355
Positive mycobacterial culture	0.74 (0.47–1.18)	0.205	0.76 (0.47–1.23)	0.265
ADRs	0.76 (0.45–1.27)	0.292	0.79 (0.46–1.34)	0.377

*OR* odds ratio, *CI* confidence interval, *BMI* body mass index, *ESR* erythrocyte sedimentation rate, *ADR* adverse drug reaction

## Discussion

This study investigated the demographic and clinical characteristics, treatment regimens, treatment outcomes, and ADRs of patients treated for MAC-PD by age group. Overall, the median number of total and parenteral drugs used did not differ across age groups. However, the long-term treatment success rate decreased with age, plummeting in patients aged ≥ 80 years. Conversely, the rate of ADRs requiring discontinuation of treatment increased with age, and the number was twice as high in patients aged ≥ 80 years than in those aged < 50 years. In multivariable analysis, age ≥ 80 years was inversely associated with worse treatment outcomes, while ADR was not associated.

The key findings of this study suggest that our treatment practice that was consistently applied to patients regardless of their age or physiological reserve may adversely affect older patients, especially in individuals aged ≥ 80 years. Contrary to our expectations, the pattern of treatment, represented by the combination and the number of drugs used, did not differ across age groups. However, ADRs approximately doubled, whereas long-term treatment success rate was halved in patients aged ≥ 80 years compared with those aged < 50 years. Taking treatment duration into consideration, the occurrence of ADRs in patients aged ≥ 80 years was three times higher than in those aged < 50 years (37.8 events per 100 person-years vs. 12.4 events per 100 person-years). These results confirm that our current clinical practice needs to be revised.

By the year 2025, South Korea, which is already an aged society, is expected to become a super-aged society where the population aged ≥ 65 years comprises > 20% of the total population [32]. In addition, there is a 50% chance that the life expectancy of South Korean women will exceed 90 years by 2030 [33]. Considering the predisposition of NTM-PD in older adults [34], these demographic changes, which are not limited to South Korea, pose new challenges for physicians managing NTM-PD. However, the treatment guidelines do not provide specific recommendations based on age groups, and studies examining treatment outcomes and ADRs in older populations have been largely absent, let alone clinical trials. Our study provides practical, novel insights into the treatment of older adults with NTM-PD.

How should we treat older patients with MAC-PD, especially those aged ≥ 80 years? In this study, older age itself was associated with worse treatment outcomes irrespective of the occurrence of ADRs. This could be explained by the higher disease severity at baseline and the presence of multiple comorbidities such as cerebrovascular disease and dementia, which complicate treatment adherence in older adults. Also, age-related physiological and pharmacokinetic changes may further reduce the efficacy of treatment [35–37]. Hence, approaches that can reduce treatment toxicity while maintaining the positive effects of treatment on quality of life [21] should be pursued in older adults with MAC-PD. For instance, reducing the intensity in terms of dosage and number of drugs used appears to be a reasonable alternative in patients with frailty and multimorbidity. In addition, since a higher proportion of patients in the older group experience gastrointestinal disorders during treatment trials than that in younger groups, avoiding combination of drugs that frequently cause such discomfort can be an option.

Consideration of frailty and functional status in older patients may provide a meaningful opportunity to design individualized treatment planning for NTM-PD, as evidence is accumulating in other medical or surgical conditions in older adults [38, 39]. A recently published study demonstrated the importance of a tailored management plan guided by geriatric assessment in cancer chemotherapy [40]. In patients with advanced cancer, chemotherapy based on a geriatric assessment intervention reduced the proportion of patients experiencing toxic effects and brought positive effects such as fewer falls and fewer medications compared with the usual care group, while not compromising survival even with lower initial dosages of chemotherapeutic agents. Further research is needed to determine the potential impact of frailty and functional status in NTM-PD treatment, and individualized algorithms could be established by integrating these geriatric conditions in the clinical decision of dosing, scheduling, and the provision of supportive or palliative care.

Our study has several limitations. First, while multivariable analyses revealed age ≥ 80 years to be an independent factor associated with ADRs and long-term treatment outcome, undocumented factors may have confounded the results. While we tried to evaluate all relevant medical data within our reach, more detailed studies incorporating comprehensive functional, cognitive, nutritional, socioeconomic, and even genomic data may lead to a more precise conclusion that goes beyond the chronological age and delineates probable mechanisms behind our study. Second, since this was a retrospective study and the termination of treatment was at the discretion of the physician on duty, physicians’ lower threshold for treatment discontinuation when ADRs occur in older patients was indistinguishable and could have biased the results. In addition, due to the retrospective design of the study, sufficient information on quality of life before and after antibiotic treatment could not be collected, making such analysis unfeasible. Third, this study was conducted at a single institution, limiting the generalizability of our findings. However, the baseline characteristics (median treatment period, 17.4 months), treatment outcomes (treatment success rate, 39.9%), and ADRs (19.7%) of our study cohort were comparable to those of previous studies. Well-designed prospective studies testing different treatment regimen combinations will be necessary in the future to find the optimal drug combination that maximizes benefit and minimizes harm in older patients.

## Conclusion

Age ≥ 80 years was an independent factor associated with ADRs and long-term treatment outcome in patients with MAC-PD. Thus, close monitoring of ADRs is required when treating older patients, and given the worse outcome and frequent ADRs associated with older individuals, tailored treatment with reduced intensity is a reasonable alternative in these patients.

## Supplementary Information

**Additional file 1.** **Supplementary Figure 1.** Treatment duration, long-term treatment success, and adverse drug reactions by age as a continuous variable.

## Data Availability

The dataset used are available from the corresponding author on reasonable request.

## Abbreviations

aOR: Adjusted odds ratio
ADR: Adverse drug reaction
BMI: Body mass index
CI: Confidence interval
ESR: Erythrocyte sedimentation rate
IQR: Interquartile range
MAC: *Mycobacterium avium* Complex
NTM: Nontuberculous mycobacteria
OR: Odds ratio
PD: Pulmonary disease
