# Supplementary material for: Old age is associated with worse treatment outcome and frequent adverse drug reaction in Mycobacterium avium complex pulmonary disease
Source: BMC Pulm Med. 2022 Jul 14;22:269. doi: 10.1186/s12890-022-02063-2 (PMC9284708; doi:10.1186/s12890-022-02063-2)
Supplement: Supplementary file 1 — Additional file 1. Supplementary Figure 1. Treatment duration, long-term treatment success, and adverse drug reactions by age as a continuous variable. [file 12890_2022_2063_MOESM1_ESM.docx]

**Old age is associated with poor treatment outcome and frequent adverse drug reaction in *Mycobacterium avium* complex pulmonary disease**

**<SUPPLEMENTARY DATA>**

Joong-Yub Kim^1*^, Na Young Kim^1,2*^, Hee-Won Jung^3^, Jae-Joon Yim^1,4^, Nakwon Kwak^1,4^

^1^Division of Pulmonary and Critical Care Medicine, Department of Internal Medicine, Seoul National University Hospital, Seoul, Republic of Korea

^2^Present address: Division of Pulmonary, Allergy, and Critical Care Medicine, Department of Internal Medicine, Hallym University Dongtan Sacred Heart Hospital, Gyeonggi-do, Republic of Korea

^3^Division of Geriatrics, Department of Internal Medicine, Asan Medical Center, University of Ulsan College of Medicine, Seoul, Republic of Korea

^4^Department of Internal Medicine, Seoul National University College of Medicine, Seoul, Republic of Korea

^*^These authors contributed equally to this manuscript

**Corresponding author:** Nakwon Kwak

Division of Pulmonary and Critical Care Medicine, Department of Internal Medicine, Seoul National University College of Medicine, 101, Daehak-ro, Jongno-gu, Seoul, 03080, Republic of Korea, E-mail: [n.kwak@snu.ac.kr](mailto:n.kwak@snu.ac.kr)

**Supplementary Figure 1. Treatment duration, long-term treatment success, and adverse drug reactions by age as a continuous variable**

**
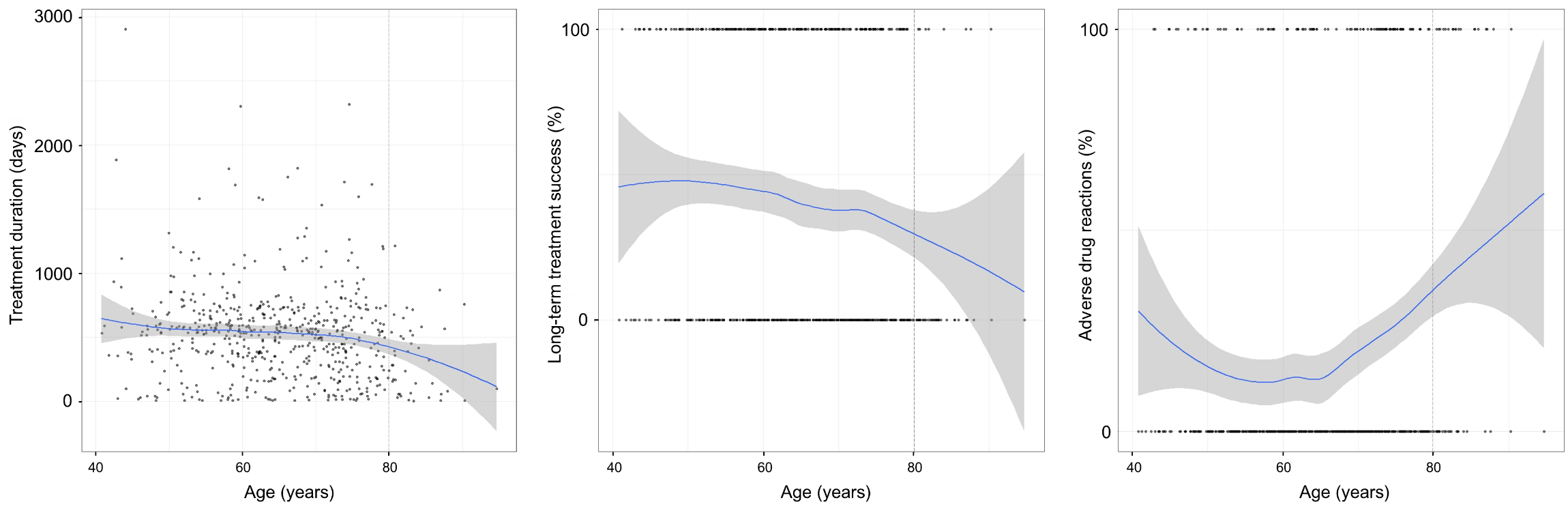
**
